# Serum testosterone and prostate cancer in men with germline *BRCA1/2* pathogenic variants

**DOI:** 10.1002/bco2.156

**Published:** 2023-01-09

**Authors:** Alexander Dias, Mark N. Brook, Elizabeth K. Bancroft, Elizabeth C. Page, Anthony Chamberlain, Sibel Saya, Jan Amin, Christos Mikropoulos, Natalie Taylor, Kathryn Myhill, Sarah Thomas, Edward Saunders, Tokhir Dadaev, Daniel Leongamornlert, Thomas Dyrsø Jensen, D. Gareth Evans, Cezary Cybulski, Annelie Liljegren, Soo H. Teo, Lucy Side, The IMPACT study collaborators and Steering Committee, Zsofia Kote‐Jarai, Rosalind A. Eeles

**Affiliations:** ^1^ Oncogenetics Team The Institute of Cancer Research London UK; ^2^ Instituto Nacional de Cancer Jose de Alencar Gomes da Silva INCA Rio de Janeiro Brazil; ^3^ Academic Urology Unit Royal Marsden NHS Foundation Trust London UK; ^4^ Clinical Biochemistry Section Royal Marsden NHS Foundation Trust London UK; ^5^ Department of Clinical Genetics Vejle Hospital Vejle Denmark; ^6^ Genetic Medicine, Manchester Academic Health Sciences Centre Central Manchester University Hospitals NHS Foundation Trust Manchester UK; ^7^ International Hereditary Cancer Center, Department of Genetics and Pathology Pomeranian Medical University in Szczecin Szczecin Poland; ^8^ Karolinska University Hospital and Karolinska Institutet Stockholm Sweden; ^9^ Cancer Research Initiatives Foundation Subang Jaya Medical Centre Selangor Darul Ehsan Malaysia; ^10^ Wessex Clinical Genetics Service Princess Anne Hospital Southampton UK

**Keywords:** androgens, biomarkers, *BRCA1/BRCA2* pathogenic variant, prostate cancer, testosterone

## Abstract

**Objectives:**

The relation of serum androgens and the development of prostate cancer (PCa) is subject of debate. Lower total testosterone (TT) levels have been associated with increased PCa detection and worse pathological features after treatment. However, data from the Reduction by Dutasteride of Prostate Cancer Events (REDUCE) and Prostate Cancer Prevention (PCPT) trial groups indicate no association. The aim of this study is to investigate the association of serum androgen levels and PCa detection in a prospective screening study of men at higher genetic risk of aggressive PCa due to *BRCA1/2* pathogenic variants (PVs), the IMPACT study.

**Methods:**

Men enrolled in the IMPACT study provided serum samples during regular visits. Hormonal levels were calculated using immunoassays. Free testosterone (FT) was calculated from TT and sex hormone binding globulin (SHBG) using the Sodergard mass equation. Age, body mass index (BMI), prostate‐specific antigen (PSA) and hormonal concentrations were compared between genetic cohorts. We also explored associations between age and TT, SHBG, FT and PCa, in the whole subset and stratified by *BRCA1/2* PVs status.

**Results:**

A total of 777 participants in the IMPACT study had TT and SHBG measurements in serum samples at annual visits, giving 3940 prospective androgen levels, from 266 *BRCA1* PVs carriers, 313 *BRCA2* PVs carriers and 198 non‐carriers. The median number of visits per patient was 5. There was no difference in TT, SHBG and FT between carriers and non‐carriers. In a univariate analysis, androgen levels were not associated with PCa. In the analysis stratified by carrier status, no significant association was found between hormonal levels and PCa in non‐carriers, *BRCA1* or *BRCA2* PVs carriers.

**Conclusions:**

Male *BRCA*1/2 PVs carriers have a similar androgen profile to non‐carriers. Hormonal levels were not associated with PCa in men with and without *BRCA1/2* PVs. Mechanisms related to the particularly aggressive phenotype of PCa in *BRCA2* PVs carriers may therefore not be linked with circulating hormonal levels.

## 1 INTRODUCTION

Accumulating evidence has shown that *BRCA2* pathogenic variants (PVs) carriers are at higher risk of developing prostate cancer (PCa), estimated to be 2.5–8.6 fold, and that their disease progression is especially accelerated.^1^, ^2^, ^3^, ^4^ Despite representing a small fraction of PCa cases (0.45%–1% and 1.2% for *BRCA1* and *BRCA2*, respectively), studies have demonstrated that these patients present with a larger proportion of high‐grade and metastatic disease.^5^ Moreover, cancer‐specific survival and metastasis‐free survival are significantly higher in non‐carriers, and carriers have a worse clinical outcome when treated with curative intent.^6^ Lifetime risks for *BRCA1* PVs carriers have been described as higher than the overall population, but lower than for *BRCA2* carriers. However, for this genetic group, the evidence is inconsistent, with relative risks ranging from 0.3 to 4.^3^, ^7^

The molecular mechanisms responsible for this aggressive disease behaviour in *BRCA2* carriers, and in particular, the clinical differences between *BRCA1* and *BRCA2* PVs carriers, are still largely unknown. *BRCA1*/*BRCA2* are tumour suppressor genes.^8^ *BRCA1* acts in multiple pathways, including DNA damage response and repair, transcriptional regulation and chromatin remodelling. *BRCA2* is associated with DNA recombination and restoration, via interactions with RAD51 and PALB2.^9^ *BRCA1* and *BRCA2* impairment results in a deficiency in repairing DNA double‐stand breaks by homologous recombination (HR). Affected cells employ mutagenic pathways to repair these lesions, culminating in genomic instability and cancer predisposition.^10^

Interestingly, patients with *BRCA1/2* mutations have different disease risks and progression patterns in two other hormonally driven cancers: ovarian and breast. In these cancers, there are also distinct clinical phenotypes between *BRCA1* and *BRCA2* PVs carriers. How these abnormalities in generic pathways translate in tissue‐specific phenotypes is unknown, and the findings in these three hormonally driven cancers may indicate that other modifiers, such as androgen exposure, may be relevant. Recent studies linked DNA‐repair mechanisms and androgen signalling.^11^, ^12^, ^13^

Many studies have interrogated the association between androgen levels and PCa risk. Low serum total testosterone (TT) levels have been associated with a higher risk of PCa^14^, ^15^ and worse pathological characteristics after surgery.^15^, ^16^, ^17^, ^18^, ^19^ Additionally, others demonstrated that low serum TT levels may have implications for the long‐term oncological outcomes, with worse 5‐year biochemical free‐survival.^20^ However, several studies have shown discordant results, such as the secondary analysis of the Reduction by Dutasteride of Prostate Cancer Events (REDUCE) and Prostate Cancer Prevention (PCPT) trials.^21^, ^22^

Due to the controversy about the role of androgens in PCa risk and to recent evidence linking DNA‐repair and androgen signalling, it is vital to explore the potential influence of hormonal levels in PCa risk in men with DNA‐repair mutations. The IMPACT study (Identification of Men with a genetic predisposition to ProstAte Cancer: Targeted screening in men at higher genetic risk and controls) is a prospective multicentre PCa screening study involving men at increased genetic risk and controls.^23^ More than 2000 participants were recruited, and the interim analyses have provided evidence favouring prostate‐specific antigen (PSA) screening in men with *BRCA2* PVs.^24^

One study described serum androgen levels in a male cohort of *BRCA1/2* PVs carriers. This study did not include men with PCa and included only 29 *BRCA2* carriers.^25^ Our objective is to interrogate whether serum levels of TT, SHBG and FT could vary by PVs status and if these levels could be associated with PCa among men enrolled in the IMPACT study, across different genetic categories.

## 2 MATERIALS AND METHODS

### 2.1 The IMPACT study

The IMPACT study methodology has been published previously.^23^, ^26^ The protocol was approved by the West‐Midlands Research and Ethics Committee in the United Kingdom (reference 05/MRE07/25) and by each participating institution's committee. Men aged 40–69 were recruited from families with *BRCA1/2* PVs. These men could enter the study if they had tested positive or negative for the PVs. Men who had a negative test for *BRCA1* were not tested for *BRCA2* mutations and vice versa. In this analysis, the control group is composed of participants who tested negative for the familial *BRCA1* or *BRCA2* PVs, in a combined control group.

Participants underwent annual PSA testing, and if the result was >3.0 ng/ml, a transrectal ultrasound guided prostate biopsy was recommended. We retrospectively reviewed data from patients participating in the IMPACT study that had at least one TT measurement, all taken previously to any PCa diagnosis or treatment.

At study entry, age and body mass index (BMI) were recorded. PSA levels were collected at baseline and prospectively. As part of study protocol, hormone levels were assessed in a subset of participants. Serum samples were obtained by venepuncture between 07:30 and 20:30 h.

### 2.2 Testosterone assays and calculations

TT measurements were based on immunoassays for most IMPACT centres, with the exception of the Denmark Centre, which employed liquid chromatography/mass spectrometry.

The free testosterone (FT) levels were calculated using the Sodergard equation using a constant albumin concentration.^27^

### 2.3 Statistical analysis

Individuals who were not diagnosed with PCa at the first screening round contributed more than one sample. TT levels can vary widely in the same individual.^28^ Therefore, instead of using a single value of TT (such as baseline, pre‐diagnostic or mean value), we included all the values available in each specific genetic cohort.

We analysed the sex hormone binding globulin (SHBG), TT and FT levels as continuous variables and TT dichotomised using a cut‐off point of 8 nmol/L (230 ng/dl), the testosterone replacement threshold for most specialty societies.^29^

Two‐way analysis of variance (ANOVA) was used to compare age and BMI at baseline between mutation carriers (*BRCA1* and *BRCA2* as separate groups) and non‐carriers, controlling for study centre, included as a factor variable.

Differences in hormonal levels (TT, SHBG and FT) among mutation carriers and non‐carriers were assessed using a nested two‐way ANOVA on log‐transformed values. Study centre was included as a factor variable, and a nested model was used to allow for repeated observations from each subject.

To explore associations between hormonal levels and PCa across PVs status, a binomial logistic regression model was used, with the covariates BMI, PSA and hormonal levels (TT, SHBG and FT), adjusted for study centre and age. All the available readings from each individual were included in the analysis. Cluster‐robust estimators were employed to relax the assumption of independent observations, thus allowing for repeated observations from each subject.

A *p*‐value of <0.05 was considered significant. All tests are two‐sided. All analyses were performed in STATA 14, College Station, TX, StataCorp LP and SPSS Version 25, IBM.

## 3 RESULTS

**FIGURE 1:**
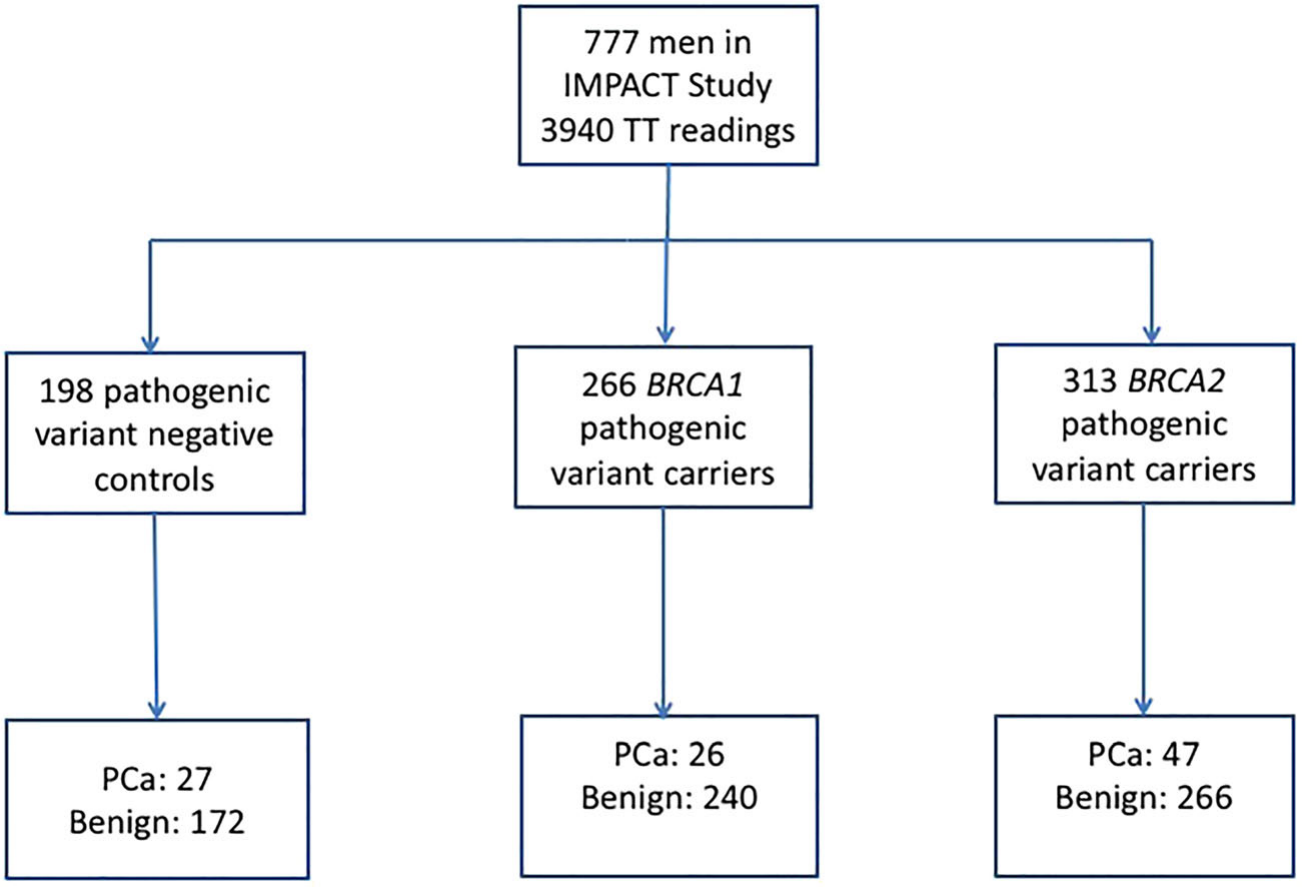
Consort diagram of study population. PCa, prostate cancer; TT, total testosterone

A total of 777 participants in the IMPACT study had at least one TT measurement (Table S1). A total of 3940 unique measurements were included. The median number of follow‐up visits per patient was 5; 198 non‐carriers, 266 *BRCA1* and 313 *BRCA2* PVs carriers were included. SHBG levels were available for 737 participants. Overall, 12.9% of the patients were diagnosed with PCa (100 out of 777), 27 non‐carriers, 26 *BRCA1* and 47 *BRCA2* PVs carriers, respectively (Figure 1).

Demographic characteristics of participants (age and BMI), baseline PSA, hormonal levels and number of follow‐up visits are shown in Table 1. *BRCA2* carriers were younger, with a median age at study entry of 51 years old (inter‐quartile range [IQR] 45–59). The median age for non‐carriers was 55 years old (IQR 47–62) and 53 years old for *BRCA1* carriers (IQR 46–60) (*p* = 0.008). BMI was higher in *BRCA2* carriers (26.67 vs. 27.16 vs. 27.53 kg/m^2^, non‐carriers, *BRCA1* and *BRCA2*, respectively, *p* = 0.02). This difference was more pronounced in patients who received a PCa diagnosis (Table S2). The number of study visits was similar between groups. We observed no differences in median baseline values of TT, SHBG and FT across the genetic categories. The majority of the samples were collected in the morning period (Table S4). There was no significant correlation between PSA and TT levels (Figure S1).

**TABLE 1:** Characteristics of subjects at baseline/first visit

	Mutation negative carriers			*BRCA1* PVs carriers	*BRCA2* PVs carriers	Total	*p*‐value^a^
	*BRCA1−*	*BRCA2−*	Total				
Subjects, *N*	103	95	*198*	266	313	777	
Age, years, median (IQR)	54 (46–61)	57 (49–64)	*55 (47–62)*	53 (46–60)	51 (45–59)	*53 (46–60)*	0.008
BMI, kg/m^2^, mean (SD)	26.87 (3.75)	26.45 (3.54)	*26.67 (3.65)*	27.16 (3.97)	27.53 (5.22)	*27.18 (4.45)*	0.020
SHBG, nmol/L, median (IQR)^b^	36 (27.00–43.50)	38.5 (29.00–55.00)	*36 (28.00–48.00)*	35 (26.20–47.00)	36 (27.00–47.00)	*36 (27.00–47.00)*	0.298
Testosterone^b^							
Total testosterone, nmol/L, median (IQR)	12.5 (10.10–15.20)	13 (10.00–16.87)	*12.8 (10.00–16.00)*	13 (9.96–16.40)	13 (10.10–16.10)	*13 (10.00–16.20)*	0.916
Free testosterone, nmol/L, median (IQR)	0.25 (0.19–3.35)	0.23 (0.18–0.28)	*0.24 (0.19–0.29)*	0.24 (0.20–0.31)	0.25 (0.20–0.31)	*0.24 (0.20–0.30)*	0.825
PSA^b^							
PSA, ng/ml, median (IQR)	0.99 (0.61–1.60)	0.96 (0.60–1.60)	*0.98 (0.60–1.60)*	0.81 (0.54–1.40)	0.83 (0.50–1.40)	*0.87 (0.56–1.50)*	0.064
Free PSA, ng/ml, median (IQR)	0.30 (0.21–0.47)	0.29 (0.21–0.43)	*0.30 (0.21–0.44)*	0.25 (0.19–0.42)	0.24 (0.18–0.39)	*0.26 (0.19–0.42)*	0.191
Calculated fraction of PSA, %, median (IQR)	25 (21–35)	29 (20–38)	*27 (20.5–37)*	29 (21–40)	31 (20–39)	*29 (21–39)*	0.212
Gleason score in cancers, *N*							
6	9	9	*18*	15	24	*57*	
3 + 4		4	*4*	7	11	*22*	
4 + 3	1	2	*3*	2	1	*6*	
8+	1	1	*2*	2	10	*14*	
Missing					1	*1*	
Total follow‐up visits, *N* (median)	528 (5)	453 (5)	981 (5)	1325 (5)	1638 (5)	3944 (5)	

Abbreviations: BMI, body mass index; IQR, inter‐quartile range; PSA, prostate‐specific antigen; PVs, pathogenic variants.

^a^ Across mutation status (negative carriers as one group, *BRCA1+*, *BRCA2+*). Two‐way analysis of variance with covariate and centre.

^b^ Test performed on log‐transformed values.

Considering all the prospective samples, we observed no differences in median values of TT, SHBG and FT across the genetic categories, in both participants with and without a PCa diagnosis (Table S3). The median TT level in the non‐carrier group was 12.6 nmol/L (IQR 9.8–15.6), 12.9 nmol/L in *BRCA1* mutation carriers (IQR 9.7–15.9) and 13.0 nmol/L in *BRCA2* mutation carriers (IQR 10.00–16.0) (*p* = 0.670). *BRCA1/2* status showed no influence on hormonal levels (Table 1 and Tables S2 and S3).

On univariate logistic regression, age was a significant predictor of PCa (odds ratio [OR] 1.085, 95% confidence interval [CI] 1.02, 1.09, *p* = 0.002). Considering the entire cohort, the OR (and 95% CI) for SHBG, TT and FT was 1.025 (0.54, 1.95), 0.95 (0.45, 2.04) and 0.87 (0.46, 1.66), respectively, all with *p* > 0.1 (Table 2). The association remained non‐significant when using TT as a dichotomous variable.

**TABLE 2:** Univariate associations between covariates and prostate cancer risk, adjusted for study centre*

	Non‐cancers (subjects (*N*), measurements (*N*))	Cancers (subjects (*N*), measurements (*N*))	OR (95% CI)	*p*‐value
Age	658 (3684)	92 (218)	1.056 (1.02, 1.09)	0.002
SHBG	626 (3497)	89 (208)	1.025 (0.54, 1.95)	0.940
Testosterone**				
Total testosterone	658 (3684)	92 (218)	0.954 (0.45, 2.04)	0.904
Free testosterone	626 (3498)	89 (208)	0.879 (0.46, 1.66)	0.692
Testosterone (<8/≥8)***				
<8	185 (418)	15 (24)	1.018 (0.47, 2.18)	0.964
≥8	641 (3266)	88 (194)		

Abbreviations: CI, confidence interval; OR, odds ratio; SHBG, sex hormone binding globulin.* Robust variance estimators to allow for sampling of subjects at multiple timepoints.** Log‐transformed values.*** Subjects can contribute data to both categories

We then assessed if the associations between androgen levels and PCa could vary accordingly with genetic status. A second regression model, stratified by *BRCA1/2* gene category and adjusted for age, mostly showed no significant associations between hormonal levels and PCa. In the non‐carriers' group, the OR for the association of TT readings and a PCa diagnosis was 2.640 (95% CI 0.20, 34.80). In *BRCA1* carriers, it was 1.339 (95% CI 0.42, 4.29), and in *BRCA2* carriers, the OR was 0.696 (95% CI 0.33, 1.47). Similar findings were observed for FT. For SHBG levels, only in *BRCA2* carriers did the association with PCa reach the level of statistical significance (OR 0.345, 95% CI 0.15, 0.77, *p* = 0.009), but this was not significant in the genetic category comparison analysis (*p*‐het 0.11; Table 3).

**TABLE 3:** Associations between androgens and prostate cancer status, stratified by *BRCA* status, adjusted for centre and age^a^

	*BRCA* status	Non‐cancers (subjects (*N*), measurements (*N*))	Cancers (subjects (*N*), measurements (*N*))	OR	(95% CI)	*p*	*p*‐het
SHBG^b^							
	Negative	158 (859)	23 (47)	1.343 (0.29, 6.27)		0.707	
	*BRCA1+*	219 (1193)	24 (56)	2.585 (0.97, 6.87)		0.057	*0.464*
	*BRCA2+*	249 (1445)	42 (105)	0.345 (0.15, 0.77)		0.009	*0.119*
Testosterone^b^							
Total testosterone							
	Negative	170 (921)	24 (53)	2.640 (0.20, 34.80)		0.461	
	*BRCA1+*	227 (1245)	25 (57)	1.339 (0.42, 4.29)		0.623	*0.633*
	*BRCA2+*	261 (1518)	43 (108)	0.696 (0.33, 1.47)		0.343	*0.330*
Testosterone (<8/≥8)^c^							
<8	Negative	49 (90)	2 (3)	1.459 (0.25, 8.49)		0.674^d^	
≥8		167 (831)	23 (50)				
<8	*BRCA1+*	69 (162)	2 (4)	2.330 (0.44, 12.20)		0.317^d^	
≥8		214 (1083)	25 (53)				
<8	*BRCA2+*	67 (166)	11 (17)	0.858 (0.30, 2.43)		0.773^d^	
≥8		260 (1352)	40 (91)				

Abbreviations: CI, confidence interval; OR, odds ratio; SHBG, sex hormone binding globulin.

^a^ Robust variance estimators to allow for sampling of subjects at multiple timepoints.

^b^ Log‐transformed values.

^c^ Subjects can contribute data to both categories.

^d^ No significant interactions.

## 4 DISCUSSION

In this work, we provide the largest report of androgen levels in a cohort of male *BRCA1/2* PVs carriers. We have found no association between *BRCA1/2* variant and baseline hormonal levels. We also investigated the association of TT, SHBG, FT and a PCa diagnosis during the IMPACT screening study. We found that pre‐diagnostic TT, FT and SHBG were not associated with PCa risk in this cohort.

Circulating androgens have a clear relationship with PCa progression, as illustrated by the therapeutic effect of androgen suppression in metastatic disease, maintained even in later stages of disease.^30^, ^31^ Serum androgens are required for the normal prostate growth and for the development of PCa, but how they act in the initial carcinogenesis is unknown, with distinct models being proposed.^32^, ^33^, ^34^, ^35^ The wide range of results in the literature is probably due to the complexity of androgen physiology and assessment. There is a significant intra‐individual variability on TT levels.^36^ In our study, only baseline BMI data were available, not allowing for adjustment for this variable in the regression analysis. At baseline, men with a *BRCA2* PV showed a higher mean BMI level. However, the observed absolute difference was small, with a narrow SD, with mean values around normal/overweight BMI classification for all genetic categories. This means that most men in the study are not in the superior extreme of BMI distribution, where a clinically relevant effect on TT levels would be expected.^37^ Additionally, most middle aged men do not experience a major change on BMI levels over a 5‐year period.^38^ Therefore, we do not expect that this could have had a major influence on the observed results.

There are limited data on androgens in men with genetic predisposition to PCa. In 2017, Goldberg et al. reported androgen levels in 87 *BRCA1/2* PVs carriers and 43 non‐carriers.^25^ They reported higher TT and free androgen index and lower prolactin levels in carriers. Our series, with a larger sample size (579 carriers and 198 non‐carriers), failed to reproduce these results. Despite showing a higher level of TT in carriers, in the Goldberg et al. study, the mean levels in carriers and controls are within the considered physiologic range and were based on a single sample from each individual. It is well known that a day‐to‐day variation within the physiologic range occurs.^36^ Most specialty societies^29^ do not recommend any hormonal intervention should be considered based on a single hormonal level. The differences in the results are probably explained by the larger sample size and the availability of multiple samples from each individual in IMPACT.

The aim of the study is looking at the association of serum androgens in a population of men at higher risk of aggressive PCa due to *BRCA* status, and a stratified analysis between low‐ and high‐grade cancer would be highly relevant. However, this study was conducted using data collected after the initial screening rounds of the IMPACT study, representing 777 participants. The number of PCa cases detected at that stage was small, not allowing for a proper statistical analysis using subgroups based on grade. The IMPACT study is ongoing, with more than 2000 participants,^24^ and the interim analysis showed higher numbers of aggressive disease in *BRCA2* PV carriers. The final analyses will allow statistical power for a follow‐up publication with stratification between low‐ and high‐grade cancer.

Our findings should not be interpreted as a definitive evidence of no interaction between the hormonal and DNA‐repair pathways as a disease modifier in *BRCA1/2* PVs carriers. There is growing evidence linking DNA damage and androgen signalling pathways in PCa.^11^ *BRCA1* and *BRCA2* are believed to act as androgen receptor (AR) coactivators. Yeh et al. demonstrated that *BRCA1* enhances transcription of AR target genes and performs an important role in androgen‐induced apoptosis by upregulating the expression of p21, because dihydrotestosterone (DHT) dramatically induces the expression of p21 in PCa cells expressing *BRCA1* and the AR.^39^ *BRCA2* has been demonstrated to associate with the AR and enhance its transcriptional activity.^40^ On the other hand, AR has been shown to regulate expression of DNA‐repair genes and related proteins. DNA‐dependent protein kinase catalytic subunit (DNAPKcs), Ku70 and Ku80, are key components of the non‐homologous end joining (NHEJ) double strand break DNA repair pathway. In a PCa model, AR was found to directly regulate DNAPKcs expression, as androgen stimulation elicited upregulation of both mRNA and protein levels of DNAPKcs.^12^

Polkinghorn et al. identified 32 DNA‐repair genes that comprise part of the complex AR‐regulated transcriptome. These genes were both induced by androgen and exhibit AR peaks in their enhancer or promoter. Many of these genes, such as *RAD51* and *CHEK1*, are involved in BRCA‐mediated DNA repair.^13^ Interestingly, the same authors also reported that the NHEJ is affected by androgen deprivation. NHEJ has been accepted as an error‐prone DNA‐repair pathway. However, recent evidence has shown that a more precise NHEJ modality exists in eukaryotic cells and that this is dependent on BRCA/DNA PKCs machinery.^9^, ^41^

In view of the evidence linking DNA‐repair and androgen signalling, showing that AR activation can trigger and enhance DNA‐repair, we postulated that a mechanism to justify the clinical behaviour in *BRCA2* PVs carriers could be a cumulative carcinogenic effect between lower TT and a deficient DNA‐repair pathway. However, we found no association between serum levels of TT, FT and PCa risk and this was not influenced by *BRCA1/2* status.

The lack of association between FT and PCa in this cohort may also be explained by the method of assessment of this variable. We used the Sodergard calculation, reliable clinically, but may not be accurate enough for the question posed here. Also, the interactions between AR signalling and DNA‐repair pathways may rely on more diverse mechanisms, and albumin–testosterone or SHBG–testosterone complexes may play an independent role.

We found that in *BRCA2* carriers, lower levels of SHBG reached statistical significance for an association with PCa in univariate analysis, but this was not sustained in the genetic group comparison. Data on association of SHBG and PCa are scarce, even when considering sporadic PCa cases.^42^ In addition to the well‐known function of binding oestrogens and androgens of SHBG, different authors proposed an independent role for SHBG in the prostate, through interaction with a specific membrane receptor, leading to activation of AMPc and increase in PSA expression and apoptosis. How this pathway could interact with the DNA‐repair mechanisms is yet to be explored.^43^

The IMPACT population represents an opportunity to address the hypothesis that androgens can lead to distinct disease behaviour in *BRCA1/2* PVs carriers. To date, it is the largest cohort recruited of *BRCA1/2* carriers, which are predisposed to aggressive disease at a younger age.^5^ Identification of mechanisms of disease progression in this group could help to understand the biological mechanisms of sporadic tumours that share the same phenotype.^10^ Secondly, due to the longitudinal design, multiple samples are collected and recorded prospectively, minimising influence of physiological variation.

The main limitation of our study is that androgen analysis is not an a priori defined hypothesis of the IMPACT study. Standardisation of sample collection and analysis can further improve future/follow‐up studies. Additionally, the levels of circulating androgens may not reflect the intraprostatic hormonal milieu.^44^ The small numbers of PCa cases could also be relevant. Finally, the inclusion of cases detected in the first screening round limit our ability to draw conclusions about the causal relationship between hormonal levels and PCa, with reverse causation being a possibility.

## 5 CONCLUSION

Men with and without *BRCA1/2* PVs have similar baseline androgen levels. We found that pre‐diagnostic TT, FT and SHBG are not relevant predictors of PCa risk and this was not affected by *BRCA1/2* status in this cohort. The mechanisms of disease could differ between these distinct genetic cohorts, and specific pathways are not fully understood. Our study suggests that circulating androgens are not associated with PCa risk in *BRCA1/2* PVs carriers and cannot explain the distinct biological behaviour of *BRCA‐*related prostate tumours.

## AUTHOR CONTRIBUTIONS

Rosalind A. Eeles had full access to all the data in the study and takes responsibility for the integrity of the data and the accuracy of the data analysis. Study concept and design: Eeles, Dias, Brook, Bancroft, Kote‐Jarai, Mikropoulos, Page, and Saya. Acquisition of data; analysis and interpretation of data; drafting of the manuscript; critical revision of the manuscript for important intellectual content; Administrative, technical, or material support: All authors. Statistical analysis: Alex, Brook, Page, Bancroft, and Kote‐Jarai. Obtaining funding: Eeles and all IMPACT collaborating sites obtained their own funding for running the study at their site. Supervision: Eeles.

## Supporting information


**Figure S1.** a. Correlation plots for the association between Log Values of Total Testosterone and log PSA. b. Correlation plots for the association between absolute Values of Testosterone and PSA

**Table S1.** Genetic status of men who had at least one TT assessment in the IMPACT study

**Table S2.** Characteristics of subjects at baseline / first visit by Genetic and Cancer status

**Table S3.** Summary of Total Testosterone, SHGB, Free testosterone, and Bio Testosterone, by Genetic Status.

**Table S4.** Samples Collection Time By Centre

## APPENDIX A THE IMPACT COLLABORATORS

### IMPACT Study Steering Committee

**PI: Prof Rosalind Eeles—**Institute of Cancer Research, London, UK

**Ms Elizabeth Bancroft—**Royal Marsden NHS Foundation Trust, London, UK

**Ms Elizabeth Page—**Institute of Cancer Research, London, UK

**Dr Mark Brook—**Institute of Cancer Research, London, UK

**Dr Zsofia Kote‐Jarai—**Institute of Cancer Research, London, UK

**Mrs Audrey Ardern‐Jones—**Royal Marsden NHS Foundation Trust, London, UK

**Prof Dr Chris Bangma—**Erasmus University Medical Center, Rotterdam, The Netherlands

**Dr Elena Castro—**Spanish National Cancer Research Center, Madrid, Spain

**Professor David Dearnaley—**Institute of Cancer Research, London, UK

**Professor Diana Eccles—**University of Southampton, Southampton, UK

**Professor Gareth Evans—**St Mary's Hospital, Manchester, UK

**Professor Jorunn Eyfjord—**University of Iceland, Reykjavik, Iceland

**Dr Alison Falconer—**Imperial College Healthcare NHS Trust, London, UK

**Professor Christopher Foster—**HCA Pathology Laboratories, London, UK

**Professor Henrik Grönberg—**University Hospital, Umea, The Netherlands

**Professor Freddie C. Hamdy—**University of Oxford, Oxford, UK

**Dr Óskar Þór Jóhannsson—**Landspitali – National University Hospital of Iceland, Reykjavik, Iceland

**Dr Vincent Khoo—**Royal Marsden NHS Foundation Trust, London, UK

**Professor Hans Lilja—**MSKCC, New York & University of Oxford, Oxford, UK

**Professor Geoffrey Lindeman—**The Walter and Eliza Hall Institute of Medical Research, Parkville Victoria, Australia

**Professor Jan Lubinski—**International Hereditary Cancer Center, Szczecin, Poland

**Dr Lovise Maehle—**Oslo University Hospital, Oslo, Norway

**Mr Alan Millner—**Royal Marsden NHS Foundation Trust, London, UK

**Dr Christos Mikropoulos**—Medway Hospital, Kent, UK

**Dr Anita Mitra—**University College London Hospitals, London, UK

**Ms Clare Moynihan—**Institute of Cancer Research, London, UK

**Dr Judith Offman—**Guy's Hospital, London, UK

**Dr Gad Rennert—**CHS National Cancer Control Center, Carmel Medical Center, Haifa, UK

**Dr Lucy Side—**Wessex Clinical Genetics Service, Southampton, UK

**Dr Mohnish Suri—**Nottingham City Hospital, Nottingham, UK

**Dr Penny Wilson—**Innovate, UK

### Retired members of the Steering Committee

**Dr Jane Melia—**University of Cambridge, Cambridge, UK

**Dr Gillian Mitchell—**Peter MacCallum Cancer Institute, Victoria, Australia

**Prof Sue Moss—**Queen Mary University of London, UK

**Prof Fritz Schroder**—Erasmus University Medical Center, Rotterdam, The Netherlands

**Prof Doug Easton**—University of Cambridge, Cambridge, UK

**Susan Peock**—University of Cambridge, Cambridge, UK

**Paul Sibley**—Siemens Healthcare Diagnostics, UK

**Reza Sharifi—**St Georges Hospital, London, UK

**Coordinating Centre, Institute of Cancer Research, London**: Rosalind Eeles, Elizabeth Bancroft, Elizabeth Page, Holly Ni Raghallaigh, Sarah Thomas Jenny Pope, Anthony Chamberlain, Romayne McMahon, Natalie Taylor, Kathryn Myhill, Denzil James, Anne Marie Borges Da Silva, Matthew Hogben, Sarah Benafif, Sibel Saya, Alex Dias.

### Australia (*more than 1 affiliation)

**Parkville Familial Cancer Centre, Peter MacCallum Cancer Centre, Melbourne, VIC**: Paul James*, Gillian Mitchell*, Sue Shanley, Kate Richardson, Joanne McKinley, Lara Petelin, Morgan Murphy, Lyon Mascarenhas, Katrina Reeve

**The Sir Peter MacCallum Department of Oncology, University of Melbourne, VIC**: Paul James, Gillian Mitchell

**Department of Urology, Peter MacCallum Cancer Centre, East Melbourne, VIC**: Declan Murphy

**Department of Urology, Flinders Medical Centre, Bedford Park, SA**: Jimmy Lam, Michael Chong

**Adult Genetics Unit, SA Pathology (at Royal Adelaide Hospital), North Adelaide, SA**: Graeme Suthers, Nicola Poplawski

**Prince of Wales Clinical School, Faculty of Medicine, UNSW, Sydney, NSW**: Robyn Ward, Katherine Tucker, Lesley Andrews, Rachel Williams

**Hereditary Cancer Clinic, Prince of Wales Hospital, Randwick, NSW**: Katherine Tucker*, Lesley Andrews*, Robyn Ward*, Rachel Williams*, Jessica Duffy

**Department of Urology, Prince of Wales Hospital, Randwick, NSW**: Richard Millard, Tom Jarvis

**St Vincent's Clinic, Sydney, NSW:** Phillip Stricker

**Familial Cancer Service, Westmead Hospital, Wentworthville, Sydney, NSW**: Judy Kirk*, Michelle Bowman

**Sydney Medical School (University of Sydney) at Westmead Millennium Institute, NSW**: Judy Kirk

**Westmead Hospital, Wentworthville, Sydney, NSW**: Manish Patel

**Familial Cancer Centre, Monash Health, Clayton, VIC**: Marion Harris, Shona O'Connell, Clare Hunt, Courtney Smyth

**Monash Medical Centre, VIC**: Mark Frydenberg

**Parkville Familial Cancer Centre, The Royal Melbourne Hospital, Parkville, VIC**: Geoffrey Lindeman*, Paul James, Catherine Morton, Kylie Shackleton

**Cancer Biology and Stem Cells Division, The Walter and Eliza Hall Institute of Medical Research, Parkville, VIC**: Geoffrey Lindeman

**Department of Medicine, The University of Melbourne, Parkville, VIC**: Geoffrey Lindeman

**Genetic Health Queensland, Royal Brisbane & Women's Hospital, Herston, QLD**: Rachel Susman, Julie McGaughran, Melanie Boon

**Genetic Services of WA, King Edward Memorial Hospital, Subiaco, WA**: Nicholas Pachter*, Sharron Townshend, Lyn Schofield

**School of Medicine and Pharmacology, University of Western Australia, Perth, WA**: Nicholas Pachter

**Hunter Family Cancer Service, Waratah, NSW**: Allan Spigelman*, Margaret Gleeson

U**niversity of New South Wales, St Vincent's Clinical School, NSW**: Allan Spigelman

**Hereditary Cancer Clinic, The Kinghorn Cancer Centre, St Vincent's Hospital, Sydney, NSW**: Allan Spigelman

**School of Biomedical Sciences and Pharmacy, University of Newcastle, NSW**: Rodney Scott

**Tasmanian Clinical Genetics Service, Hobart, TAS**: David Amor*, Paul James, Jo Burke, Briony Patterson

**Murdoch Children's Research Institute, Parkville, VIC**: David Amor

**Department of Paediatrics, University of Melbourne, Parkville, VIC**: David Amor

**Mater Private Hospitals, QLD**: Peter Swindle

**Victorian Cancer Biobank, Carlton, VIC**

**Pathwest (Clinical Trials Lab), WA**

**Pathology Queensland (Central Laboratory; Health Support Queensland), QLD Department of Health, Herston, QLD**

**Department Gynaecological Oncology Laboratory, Westmead Hospital, Centre for Cancer Research, NSW**

### Canada

**McGill University, Montreal**: William Foulkes, Zoulikha Rezoug, Leila Feng, Talia Boshari, Nassim Taherian, Armen Aprikian, Marie Jeanjean

### Denmark

**Department of Clinical Genetics, Vejle Hospital, Vejle**: Thomas Dyrsø Jensen, Karina Rønlund

**Department of Urology, Vejle Hospital, Vejle**: Palle Osther, Majbritt Kure Tondering

**Odense University Hospital, Odense**: Anne‐Marie Gerdes

### Germany

**Center of Familial Breast and Ovarian Cancer, University Hospital of Cologne, Cologne**: Rita Schmutzler, Kerstin Rhiem, Kerstin Luedtke‐Heckenkamp, Petra Wihler, Nicole Ochsendorf, Kerstin Fiddike, Natalie Herold

**University Hospital Dresden**: Dr K Kast, S Zastrow, C Griebsch

### Iceland

**University Hospital of Iceland, Reykjavik**: Oskar Johannsson, Vigdis Stefansdottir

### India

**Tata Memorial Centre, Mumbai**: Vedang Murthy, Rajiv Sarin, Pradnya Kowtal

### Ireland

**Mater Private Hospital, Dublin**: David Gallagher, Richard Bambury, Michael Farrell, Fergal Gallagher Ingrid Kiernan, Martina Smith, Siobhan Warren, Aisling Cahill, Verena Murphy, Catherine Fleming, Elizabeth Morrin, Michael Farrell

### Israel

**The Genetic Institute, The Gastroenterolgy Institute and the Urology Department, Kaplan Medical Centre, Rehovot**: Rakefet Chen‐Shtoyerman, Alon Basevitch, Dan Leibovici, Ehud Melzer and Sagi Josefsberg Ben‐Yehoshua

**Chaim Shema Medical Center, Tel‐Hashomer**: Eitan Friedman

### Italy

**Istituto Nazionale dei Tumori, Milan**: Nicola Nicolai, Paolo Radice, Riccardo Valdagni, Tiziana Magnani, Fabiana Zollo, Mario Catanzaro, Margherita Sorrentino, Simona Gay, Marco Vitellaro

### Malaysia

**Cancer Research Initiatives Foundation, Subang Jaya Medical Centre, Selangor Darul Ehsan**: Soo Hwang Teo, Hui Meng Tan, Sook‐Yee Yoon

**University of Malaya, Kuala Lumpur**: Soo Hwang Teo, Meow Keong Thong

### The Netherlands

**STOET**: **Stichting Opsporing Erfelijke Tumoren, Leiden**: Hans Vasen

**Leiden University Medical Centre, Leiden**: Christi van Asperen, Janneke Ringelberg‐Borsboom, Helen van Randeraad

**Radboud University Medical Center**: Lambertus A. (Bart) Kiemeney, Wendy van Zelst‐Stams

**University Medical Center Utrecht**: Margreet G.E.M. Ausems, Rob B. Van der Luijt

**Academic Medical Center, Amsterdam**: Theo van Os

**Netherlands Cancer Institute, Amsterdam**: Mariëlle W.G. Ruijs

**VU University Medical Center, Amsterdam**: Muriel A. Adank

**Erasmus Medical Center, Rotterdam**: Rogier A. Oldenburg

**University Hospital Maastricht**: A. (Paula) T.J.M. Helderman‐van den Enden

**University Medical Centre Groningen**: Jan C. Oosterwijk

*List of Urologists

### Norway

**Oslo University Hospital**: Lovise Mæhle, Eli Marie Grindedal, Eldbjørg Hanslien

**Akershus University Hospital**: Karol Axcrona

### Poland

**International Hereditary Cancer Centre, Szczecin**: Cezary Cybulski, Jan Lubinski, Dominika Wokolorczyk

### Portugal

**Portuguese Oncology Institute, Porto**: Manuel Teixeira, Sofia Maia, Marta Cardoso, Ana Peixoto, Rui Henrique, Jorge Oliveira, Nuno Gonçalves, Luís Araújo, João Paulo Souto, Pedro Nogueira

### Slovakia

**National Cancer Institute, Bratislava**: Denisa Ilencikova, Lucia Copakova

### Slovenia

**Institute of Oncology, Ljubljana**: Janez Zgajnar, Mateja Krajc, Alenka Vrecar

### Spain

**Hereditary Cancer Program, ICO‐IDIBELL (Bellvitge Biomedical Research Institute, Catalan Institute of Oncology), CIBERONC, Barcelona, Spain**: Mónica Salinas, Gabriel Capella, Ignacio Blanco

**Hospital de Sant Pau, Barcelona**: Teresa Ramón y Cajal, Nuria Calvo Verges, Josefina Mora, Joan Palou, Consol López, David Fisas, Alexandra Gisbert

**Hospital Vall d'Hebron, Barcelona**: Judith Balmaña, Neus Gadea, Juan Morote

### Sweden

**Karolinska Univesity Hospital, Stockholm**: Annelie Liljegren, Marie Hjälm ‐ Eriksson, Karl‐Johan Ekdahl, Stefan Carlsson

### United Kingdom

**Royal Marsden NHS Foundation Trust**: Angela George, Zoe Kemp, Jennifer Wiggins, Cathryn Moss, Lizzie Verdon, Vincent Khoo, Nicholas Van As, Alan Thompson, Chris Ogden, Christopher Woodhouse, Pardeep Kumar, Declan Cahill

**Manchester Regional Genetics Service, Manchester**: D Gareth Evans, Jeanette Rothwell, Karen Tricker, Barbara Bulman

**Wessex Clinical Genetics Service, Southampton**: Lucy Side, Diana Eccles, Tessy Thomas, April Ruiz, Darran Ball, Oliver Jones, Catherine Mercer, Donna McBride, Philandra Costello Gillian Wise, Allison Pearce, Victoria Sands, Audrey Torokwa

**East Anglian Regional Genetics Service, Cambridge**: Marc Tischkowitz, Amy Taylor, Vincent Gnanapragasam, Barbara Newcombe, Joan Paterson, Virginia Clowes

**Oxford Regional Genetics Service, Oxford**: Lisa Walker, Dorothy Halliday, Emma Stobie, Helen Purnell, Barbara Stayner, Denise, Fleming‐Brown, Freddie Hamdy, Heather Chalinor, Venessa Miller, Kathryn Saunders

**South West Thames Regional Genetics Service, London**: Katie Snape, Helen Hanson, Merrie Manalo, Darshna Dudakia, Audrey Dearing, Shirley Hodgson, Glen Brice, Tessa Homfray, Carrie Hammond, Elizabeth Winchester, Mark Mencias, Siti Ismail, Sally Goff

**Peninsula Clinical Genetics Service, Exeter**: Carole Brewer, Linda Park, Joanna Ireland, Alison Potter, Caroline Renton, Anne Searle, Kathryn Hill, Selina Goodman, Lynda Garcia, Gemma Devlin, Sarah Everest, Maria Nadolski, Debbie Fuller, Catherine Grey, Melanie Hutchings, Karin Gupwell, Maggie Thomas, Matilda Bradford, Sandra Cookson, Lisa Adams

**Northern Clinical Genetics Service, Newcastle**: Ashraf Azzabi, Irene Jobson, Edgar Paez, Alex Henderson, Fiona Douglas

**South West Regional Genetics Service, Bristol**: Alan Donaldson, Amy Watford, Sue Tomkins

**South East Thames Regional Genetics Service, Guy's Hospital London**: Louise Izatt, Vishakha Tripathi, Michelle Weston, Kathrine Hilario, Merrie Manalo, Gabriella Pichert, Chris Jacobs, Caroline Langman Mark Mencias, Anjana Kulkarni, Bianca De Souza, Clare Turnbull, Alice Youngs, Cecilia Compton

**North West Thames Regional Genetics Service, Harrow**: Angela Brady, Virginia Clowes, Huw Dorkins, Falak Arshad, Athalie Melville, Monika Kosicka‐Slawinska, Carole Cummings, Vicki Kiesel, Marion Bartlett, Kashmir Randhawa, Natalie Ellery, Cheryl Sequeira, Samia Sakuma, Demetra Georgiou, Toyin Adeoye

**North East Thames Regional Genetics Service, NE Thames**: Munaza Ahmed, Alison Male, Jana Gurasashvili, Kate Simon, Katie Rees, Cecilia Compton, Lizzie Tidey, Laura Coulier, Camila Gabriel, Ellen Quinn, Nita Solanky

**Sheffield Regional Clinical Genetics Department, Sheffield**: Jackie Cook, Jennifer Davies, Pauline Bayliss, Louise Patterson, Stuart Ingram, Kelsey Armitage, Alice Howell

**Academic Urology Unit, Sheffield**: Derek Rosario, Clare Ward, James Catto, Joanne Howson

**West Midlands Regional Clinical Genetics Service, Birmingham**: Kai‐Ren Ong, Jonathan Hoffman, Camilla Huber, Wayne Glover, Farah Islam, Saira Ali, Lucy Burgess, Rachel Hart, Cyril Chapman, Tricia Heaton, Trevor Cole

**West of Scotland Genetics Service, Glasgow**: Rosemarie Davidson, Mark Longmuir, Cathy Watt, Alexis Duncan, Nicola Bradshaw, Lesley Snadden, Jennifer Gorrie

**Leicester Royal Infirmary**: Julian Barwell, Roger Kockelbergh, Shumi Mzazi, Charlotte Poile, Mandy LeButt Ayisha Sattar, Beckie Kaemba, Zahirah Sidat, Nafisa Patel, Kas Siguake, Amy Branson

**North Cumbria University Hospitals Trust**: Alex Henderson, Angela Birt, Una Poultney, Nkem Umez‐Eronini, Jaswant Mom, Beverley Wilkinson

**Royal Liverpool Children's Hospital, Liverpool**: Lynn Greenhalgh, Michaela Davies, Rachael O'Keefe, Anne Johnstone, Gillian Roberts, Anthony Woodward, Vivienne Sutton

**Royal Liverpool and Broadgreen Hospital NHS Trust, Liverpool**: Philip Cornford, Nicola Bermingham Katy Treherne, Julie Griffiths, Pembe Yesildag

**Derriford Hospital, Plymouth**: Carole Brewer, Lyn Cogley, Hannah Gott, Maria Brennan, Natale Salvatore, Sue Freemantle

**Urology Department, New Cross Hospital, Wolverhampton**: Peter Cooke, Vanda Carter, Anna Grant, Claire Lomas, Jason Rogers

### United States

**NorthShore University HealthSystem, Evanston**: Brian Helfand, Elena Genova, Christina Selkirk, Peter Hulick, Wendy Rubinstein, Charles Brendler, Michael McGuire, Karen Kaul, Daniel Shevrin, Scott Weissman, Anna Newlin, Kristen Vogel, Shelly Weiss

**Huntsman Cancer Institute, university of Utah Health, Salt Lake City, Utah**: Saundra Buys, David Goldgar, Karen O'Toole, Tom Conner, Vickie Venne, Robert Stephenson, Christopher Dechet

**University of Pennsylvania, Philadelphia**: Susan Domchek, Jacquelyn Powers

**University of Texas, MD Anderson Cancer Center, Texas**: Banu Arun, Sara Strom, John W. Davis, Yuko Yamamura

**Fox Chase Cancer Center**: Elias Obeid, Lisa Bealin, Loretta Bagden, Sue Montgomery, Veda Giri, Laura Gross

**University of Michigan**: Kathy Cooney, Elena Stoffel, Linda Okoth

### *The Netherlands Urologists

M. Asselman—Medisch Centrum Twente loc. Enschede, Haaksbergerstraat 55, 7513 ER Enschede

H.P. Beerlage—Jeroen Bosch ZH, Henrik Dunantstraat 1, 5223 GZ Den Bosch

E.R. Boevé—St. Franciscus Gasthuis, Kleiweg 500, 3045 PM Rotterdam

C.A. Bout—Isala Kliniek Wezenlanden, Groot Wezenland 20, 8011 JW Zwolle

G. Commelin—Zuiderzee ZH, Ziekenhuisweg 100, 8233 AA Lelystad

E.B. Cornel—ZGT Twente, Burgemeester Hobusstraat 56, 6031 VA Nederweert

R.J.A.M. Davits—Tweesteden Ziekenhuis, Dr. Deelenlaan 5, 5042 AD Tilburg

J.A.P.M. de Hond—Andros Mannenkliniek Arnhem, Mr. E.N. van Kleffenstraat 56 842 CV Arnhem

G.A. Dijkman—Andros Mannenkliniek Maastricht, Parkweg 29, 6212 XN Maastricht

J.G. Fernandes—Groene Hart ZH Gouda, Bleulandweg 10, 2803 HH Gouda

K.B.J. Fransis—Bravis ZH, Boerhaavelaan 25, 4708 AE Roosendaal

A.D.H. Geboers—Slingerland ZH, Kruisbergseweg 25, 7009 BL Doetinchem

R. Gilhuis—Beatrix ZH, Banneweg 57, 4204 AB Gorinchem

J.J.P. Jaspars—Adm de Ruyter ZH, 's‐Gravenpolderseweg 114, 4462 RA Goes

I.J. Jong—UMCG, Hanzeplein 1, 9700 RB Groningen

M.M.G.J. Jongen‐Huinink—Refaja Ziekenhuis Stadkanaal, Boerhaavestraat 1, 9501 HE Stadskanaal

H.F.M. Karthaus—CWZ Nijmegen, Weg door Jonkerbos 100, 6532 SZ Nijmegen

P.J.M. Kil—St. Elisabeth ZH. Hilvarenbeekseweg 60, 5022 GC Tilburg

P.E. Kleingeld—Bethesda ZH Hoogeveen, Dr. G. A. Amshoffweg 1, 7909 AA Hoogeveen

W.J. Kniestedt—St. Jansdal, St. Jansdal Harderwijk, Wethouder Jansenlaan 90, 3844 DG Harderwijk

B.C. Knipscheer—Ropken Zweers ZH, J. Weitkamplaan 4a, 7772 SE Hardenberg

R.F. Kropman—Haga ZH loc Leyweg, Leuweg 275, 2545 CH Den Haag

M.I. Lampe—Med. Centrum Leeuwarden, Henri Dunantweg 2, 8934 AD Leeuwarden

S.I. Langbein—Zaans Medisch Centrum, Kon Julianaplein 5, 1502 DV Zaandam

J.W.H.M. Langeveld—St. Jans Gasthuis Weert, Vogelsbleek 5, 6001 BE Weert

J.A.F. Leenarts—Medisch Centrum Haaglanden, Burg Banninglaan 1, 2262 BA Leidschendam

M.R. Leter—West Fries Gasthuis, Maelsonstraat 3, 1624 NP Hoorn

M.T.W.T. Lock—UMCU, Heidelberglaan 100, 3584 CX Utrecht

A.H.P. Meier—Viecuri MC, Tegelseweg 210, 5912 BL Venlo

B. Meijer—Flevo ZH, Hospitaalweg 1, 1315 RA Almere

G.J. Molijn—ZGT Twente Almelo, Zilvermeeuw 1, 7609 PP Almelo

P.F.A. Mulders—UMCN St. Radboud ZH, Geert Grooteplein‐Zuid 10, 6525 GA Nijmegen

N. Naderi—Andros Mannenkliniek, Bezuidenhoutseweg 1, 2594 AB Den Haag

M.A. Noordzij—Spaarne ZH, Spaarnepoort 1, 2134 TM Hoofddorp

J.W. Noordzij—Amstelland Ziekenhuis, Laan van Heelende Meesters 8, 1186 AM Amstelveen

F.P.P.M. Pernet—Maxima MC, Ds. Th. Fliednerstraat 1, 5631 BM Eindhoven

E.P.M. Roos—Antonius ZH Sneek, bolswarderbaan 1, 8601 ZK Sneek

M.J. Schaaf—Boven IJ ZH, Statenjachtstraat 1, 1034 CS Amsterdam

R.J.H. Schaafsma—Wilhelmina ZH Assen, Europaweg Zuid 1, 9401 RK Assen

G.A.H.J. Smits—Alysis zorggroep Rijnstate, Wagnerlaan 55, 6815 AD Arnhem

S. Smorenburg—Nij Smellinghe, Compagnonsplein 1, 9202 NN Drachten

J.R. Spermon—Diakonessenhuis Utrecht, Bosboomstraat 1, 3582 KE Utrecht

G. van Andel—Onze Lieve Vrouwen Gasthuis, Oosterpark 9, 1091 AC Amsterdam

E. van Boven—Maasziekenhuis, Dr. Kopstraat 1, 5835 DV Beugen

C. van de Beek—Academisch ZH Maastricht, Postbus 5800 6202 AZ Maastricht

I.W. van der Cruijsen‐Koeter—Beatrix ZH Gorinchem, Banneweg 57, 4204 AA Gorinchem

H.G. van der Poel—NKI/AVL, Plesmanlaan 121, 1066 CX Amsterdam

O.B. van Vierssen Trip—Gelderse Vallei, Willy Brandlaan 10, 6716 RP Ede

P.D.J. Vegt—Rijnland ZH, Simon Smitweg 1, 2553 GA, Leiderdorp

A.P.M. Verhulst—Bernhoven ZH loc OSS, Burg. De Kuijperlaan 7, 5461 AA Veghel

J. Verlind—Medisch Centrum Alkmaar, Wilhelminalaan 12, 1815 JD, Alkmaar

P. Vijverberg—St. Antonius ZH, Koekoekslaan 1, 3435 CM Nieuwegein

P.A. Wertheimer—Alb. Schweitzer ZH, Albert Schweizerplaats 25, 3318 AT Dordrecht

B.P. Wijsman—Tweesteden Ziekenhuis, Dr. Deelenlaan 5, 5042 AD Tilburg

J.V. Zambon—Laurentius ZH Roermond, Mgr. Driessenstraat 6, 6040 AXE Roermond
