# Supplementary material for: Serum testosterone and prostate cancer in men with germline BRCA1/2 pathogenic variants
Source: BJUI Compass. 2023 Jan 9;4(3):361–73. doi: 10.1002/bco2.156 (PMC10071088; doi:10.1002/bco2.156)
Supplement: Supplementary file 1 — Figure S1. a. Correlation plots for the association between Log Values of Total Testosterone and log PSA. b. Correlation plots for the association between absolute Values of Testosterone and PSA [file BCO2-4-361-s003.docx]

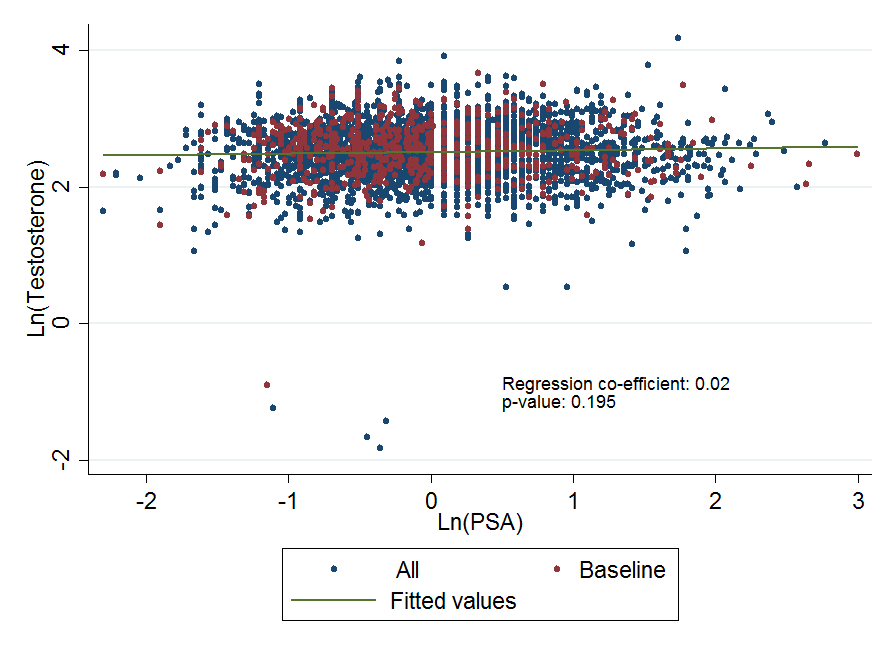


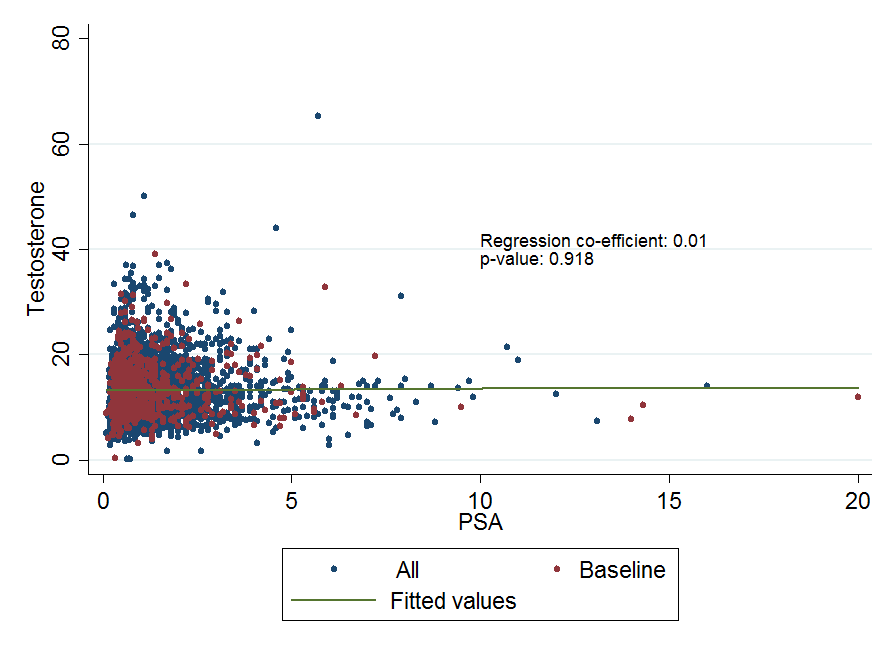


Figure 1.a.S. Correlation plots for the association between Log Values of Total Testosterone and log PSA

Fig.1.b.S - Correlation plots for the association between absolute Values of Testosterone and PSA
